# Various roles of heme oxygenase-1 in response of bone marrow macrophages to RANKL and in the early stage of osteoclastogenesis

**DOI:** 10.1038/s41598-018-29122-1

**Published:** 2018-07-17

**Authors:** Urszula Florczyk-Soluch, Ewelina Józefczuk, Jacek Stępniewski, Karolina Bukowska-Strakova, Mateusz Mendel, Monika Viscardi, Witold Norbert Nowak, Alicja Józkowicz, Józef Dulak

**Affiliations:** 1 Department of Medical Biotechnology, Faculty of Biochemistry, Biophysics and Biotechnology, Jagiellonian University, Krakow, Poland; 2 Kardio-Med Silesia, Zabrze, Poland; 3 Department of Clinical Immunology, Institute of Pediatrics, Jagiellonian University Medical College, Krakow, Poland

## Abstract

Heme oxygenase-1 (HO-1; encoded by *Hmox1*), a downstream target of the Nrf2 transcription factor, has been postulated to be a negative regulator of osteoclasts (OCLs) differentiation. Here, we further explored such a hypothesis by examining HO-1 effects in different stages of osteoclastogenesis. We confirmed the inhibition of the expression of OCLs markers by Nrf2. In contrast, both the lack of the active *Hmox1* gene or HO-1 silencing in OCLs precursor cells, bone marrow macrophages (BMMs), decreased their differentiation towards OCLs, as indicated by the analysis of OCLs markers such as TRAP. However, no effect of HO-1 deficiency was observed when HO-1 expression was silenced in BMMs or RAW264.7 macrophage cell line pre-stimulated with RANKL (considered as early-stage OCLs). Moreover, cobalt protoporphyrin IX (CoPPIX) or hemin, the known HO-1 inducers, inhibited OCLs markers both in RANKL-stimulated RAW264.7 cells and BMMs. Strikingly, a similar effect occurred in HO-1^−/−^ cells, indicating HO-1-independent activity of CoPPIX and hemin. Interestingly, plasma of HO-1^−/−^ mice contained higher TRAP levels, which suggests an increased number of bone-resorbing OCLs in the absence of HO-1 *in vivo*. In conclusion, our data indicate that HO-1 is involved in the response of bone marrow macrophages to RANKL and the induction of OCLs markers, but it is dispensable in early-stage OCLs. However, *in vivo* HO-1 appears to inhibit OCLs formation.

## Introduction

Osteoclasts (OCLs) are multinucleated myeloid cells crucial for constant bone remodelling because of their bone-resorbing activity. Excessive bone resorption coming from increased number and activity of OCLs lies at the root of most adult skeletal diseases including osteoporosis, periodontal disease, rheumatoid arthritis, multiple myeloma and metastatic cancers^1^.

Osteoclastogenesis is induced by the receptor activator of nuclear factor κB ligand (RANKL) after its binding to the RANK receptor^2–4^. In parallel, the activation of the c-fms receptor by macrophage colony-stimulating factor (M-CSF) provides the survival signal^5,6^. RANKL binding enables recruitment of adaptor molecules such as TRAF6^7^. TRAF6 activates NF-κB^8,9^, which is essential for the initial induction of nuclear factor of activated T cells, cytoplasmic 1 protein (NFAT-c1)^10,11^. NFAT-c1 activated by calcium signalling is auto-amplified after binding to its own promoter and an action of c-Fos^12^. NFAT-c1 induces the expression of OCLs-specific genes such as tartrate-resistant acid phosphatase (TRAP), cathepsin K or integrin β3^13^. In addition, RANKL action transiently increases the level of reactive oxygen species (ROS) via currently assumed RANK/TRAF6/Rac1/Nox signalling cascade^14–17^. ROS are considered as intra-cellular signalling molecules most probably targeting pro-inflammatory NF-κB pathway and promoting OCLs formation^18,19^. On the other hand, oxidative stress conditions force cells to impel protective mechanisms, which, however, are thought to be attenuated during osteoclastogenesis to secure ROS signalling^17^. Thus, such pathways are expected to be osteoclastogenic regulators of potential therapeutic significance for skeletal diseases.

Nuclear factor E2-related factor 2 (Nrf2) transcription factor represents one of the critical cytoprotective pathways controlling detoxifying, antioxidant and anti-inflammatory agents including heme oxygenase-1 (HO-1)^20,21^. Nrf2/HO-1 axis was shown to inhibit NF-κB signaling^22^. In addition, a growing body of evidence indicates a role of HO-1 in cell differentiation as shown for endothelial progenitors^23^, myoblasts^24^, erythroid progenitors^25^ or osteoblasts^26,27^. Recent studies have already suggested an inhibitory effect of both HO-1^28–32^ and Nrf2^33,34^, on osteoclastogenesis. HO-1 deficiency was shown to decrease bone density during bone remodelling *in vivo* mainly due to increased osteoclastogenesis and bone resorption^30^. Importantly however, deletion of HO-1 in the myeloid lineage attenuated the ability of myeloid progenitors to differentiate toward macrophages^35^. In addition, HO-1 with its active products was shown to regulate activation, proliferation, and survival of mature macrophages^35^. Thus, since HO-1 seems to be important for myeloid cell differentiation and macrophage function but has antioxidant and anti-inflammatory potential, its role in osteoclastogenesis might be more complex and may depend on the stage of the process.

Here we showed that while HO-1 deficiency in OCLs precursors diminishes differentiation in response to RANKL, it is dispensable in RANKL-pre-stimulated cells considered as early-stage OCLs. Thus, HO-1 seems to mediate the response of OCLs precursors to RANKL and induction of OCLs markers but is dispensable in early-stage OCLs. *In vivo*, the advantage of the inhibitory effect of HO-1 on osteoclastogenesis might be concluded. Inhibition of the expression of OCLs markers by Nrf2 was verified and confirmed.

## Materials and Methods

### Reagents

Recombinant human M-CSF and recombinant human RANKL were obtained from Sigma-Aldrich and were dissolved in water containing 0.1% BSA to a concentration of 10 μg/ml. CoPPIX and SnPPIX were purchased from Frontier Scientific, while hemin was obtained from Calbiochem and all were prepared as 10 mM stocks in DMSO or 100 mM NaOH. Sulphoraphane was purchased from Sigma-Aldrich and prepared as 5 mM stock in DMSO.

### Animals and care

All animal work was approved by the Local Ethical Committee for Animal Research at the Jagiellonian University (license no 86/2011). HO-1 (*Hmox1*) knockout (HO-1^−/−^, C57BL/6 × FVB) and wild type (HO-1^+/+^) mice, and Nrf2 (*Nfe2l2*) knockout (Nrf2^−/−^, C57BL/6) and wild type (Nrf2^+/+^) mice aged 2–4 months (males and females) were used for isolation of bone marrow cells or plasma. Genotypes were verified by PCR. All experiments were performed according to approved guidelines and regulations.

### Bone marrow and plasma isolation

Mice were sacrificed with 5 mg/ml ketamine and 2 mg/ml xylazine solution (10 μl per gram of b.w.). Blood was collected by direct heart puncture with a syringe containing 25 µl of heparin solution (1000 U/ml, Polfa) and centrifuged (10 min, 800 × g, 4 °C). Plasma was collected to new microcentrifuge tubes.

Bone marrow (BM) was isolated from tibial and femoral bones of euthanized mice. The marrow cavity was flushed out with α-MEM medium (Lonza) supplemented with 10% fetal bovine serum (FBS), 100 U/ml penicillin and 100 μg/ml streptomycin (α-MEM CM) using a sterile 20-gauge needle. A single cell suspension of bone marrow cells (BMCs) obtained by pipetting was centrifuged (5 min, 100 × g, 4 °C), washed with PBS, resuspended in α-MEM CM and counted using Muse™ Count and Viability Assay Kit and Muse Cell Analyzer (Merck Millipore).

### Cell culture and treatment

BMCs from HO-1^−/−^, Nrf2^−/−^ and wild type counterparts and murine RAW264.7 macrophages cell line were cultured in α-MEM CM, in the incubators with standardized parameters: 37 °C, 5% CO_2_ and 95% humidity.

Bone marrow macrophages (BMMs) or RAW264.7 cells were used as OCLs precursors. RANKL-stimulated OCLs precursors were considered as early-stage OCLs. Three alternative experimental settings of the culture of primary cells were used (Supplementary Fig. S1). BMCs-derived bone marrow macrophages (BMMs), BMCs-derived replated BMMs and nonadherent BMCs (nBMCs) -derived replated BMMs were used as OCLs precursors for differentiation towards OCLs. Briefly, total BMCs were cultured for 3 days at high dose (100 ng/ml) of M-CSF (used to obtain BMCs-derived BMMs) to stimulate the proliferation of macrophages without growth of stromal cells^36^. Alternatively, after overnight incubation of BMCs in the presence of 50 ng/ml M-CSF, nBMCs were harvested to culture stroma-free bone marrow cells. After 3 days the adherent cells were harvested as nBMCs-derived BMMs^29^.

To induce OCLs differentiation BMCs-derived BMMs were directly stimulated with RANKL (50 ng/ml or 100 ng/ml) in the presence of 100 ng/ml M-CSF. Alternatively, BMCs-derived BMMs and nBMCs-derived BMMs were replated (detached using Accutase, centrifuged for 5 min at 100 × g, counted and seeded) and cultured in the presence of 50 ng/ml RANKL and 30 ng/ml M-CSF. Where indicated sulphoraphane (2.5 μM) was used for Nrf2 activation. After 3 days of incubation with M-CSF and RANKL TRAP staining and TRAP ELISA were performed and OCLs markers were analyzed by qPCR. Specifically, for the staining of actin structures nBMCs-derived replated BMMs were cultured with 100 ng/ml RANKL for 5 days (in the presence of 30 ng/ml M-CSF).

To examine the effect of pharmacological inducers/inhibitor of HO-1, RAW264.7 were cultured with 50 ng/ml RANKL and 25 μM CoPPIX/hemin/SnPPIX or NaOH as a vehicle. After 9 and 48 h of incubation OCLs markers were analyzed by qPCR. OCLs precursors (nBMCs-derived BMMs) were replated and cultured with 30 ng/ml M-CSF and 50 ng/ml RANKL in the presence of 5, 15 and 25 μM CoPPIX or 5, 15 and 25 μM hemin or DMSO as a vehicle. After 3 days of incubation OCLs markers were analyzed by qPCR. BMCs-derived BMMs were stimulated with 100 ng/ml M-CSF and 100 ng/ml RANKL in the presence of 25 μM CoPPIX or 25 μM hemin or DMSO as a vehicle. After 3 days of incubation TRAP staining was performed.

### FACS analysis

To assess what is the percentage of strictly defined monocytes and macrophages among the population of BMCs and BMMs, referred to as OCLs precursors, FACS analysis was performed. A single cell suspension of BMCs (1 × 10^6^) and/or nBMCs-derived BMMs (4 × 10^5^) were centrifuged (5 min, 700 × g, RT), washed with PBS, and used for FACS analysis. For detection of macrophages and monocytes a mixture of the following anti-mouse antibodies against: CD45-APC-eFluor 780, F4/80-APC, MHCII-PE-Cy7, Ly6C-PerCP-Cy5.5 (Thermo Fisher Scientific), CD11b-PE-CF594 and Ly6G-BV605 (BD Horizon) (0.6 µg of each antibody per sample) was added for 15 min at 4 °C in a final volume of 100 µl of appropriate buffer depending on protocol used. For detection of apoptotic cells, proliferating cells and ROS production, respectively, TACS Annexin V-FITC Apoptosis Detection Kit (Trevigen), antibody against Ki67-AlexaFluor 488 (BD Pharmingen) or Cell ROX Green Reagent (Thermo Fisher Scientific) were used in combination with DAPI (0.2 μg/ml). After incubation with dyes and antibodies, cells were washed with PBS, centrifuged (5 min, 700 × g, RT), resuspended in 350 μl of 2% FBS in PBS and analysed with the flow cytometer (LSR Fortessa, BD) using FACSDiva v8.1 software.

On the BD LSR Fortessa green fluorescent dyes (FITC, AlexFluor488, Cell ROX Green Reagent) as well as PerCP-Cy5.5 were excited by a 50 mW 488 nm blue laser and emitted light was collected using 530/30 BP filter for all green dyes and by 675/20 BP filter for the latter dye. PE, PE-CF594 and PE-Cy7 were excited by 50 mW 561 nm green-yellow laser and their emission spectra were collected with 582/15 BP, 610/20 BP and 780/60 BP filters. APC and APC-eFluor780 were excited by a 40 mW 640 nm red laser and their emission was collected with 670/30 BP and 780/60 BP filters respectively. Whereas DAPI and BV605 were excited by a 50 mW 405 nm violet laser and emitted light was collected with 450/40 BP and 610/20 BP filters, respectively.

#### Analysis and gating strategy

In the first step doublets exclusion was done based on elimination of events with increased FSC-Width values. To assess specificity of Annexin-V, Ki67 staining and ROS production in populations of interest, FMO controls were used. As in whole bone marrow samples internal control populations are available, a positivity of remaining parameters was defined by in-sample cell controls which do not express the antigen (i.e., mature, cross-lineage cells gated in plots with CD45 and/or cross-lineage markers). This approach is said to be one of the most appropriate control measures due to the exposure of all populations to identical condition^37^.

Monocytes were identified based on Ly6G negativity, CD45 positivity, high Ly6C and CD11b expression, and low to negative MHCII and F4/80 expression (CD45^+^Ly6G^−^Ly6C^+^CD11b^+^MHCII^low/−^F4/80^low/−^). Of note, the term “monocytes” is only used in case of BMCs population precisely defined as CD45^+^Ly6G^−^Ly6C^+^CD11b^+^ MHCII^low/−^F4/80^low/−^. The term “BMCs” is referred to the whole population of cells obtained after bone marrow isolation.

Macrophages were identified based on positivity for CD45, F4/80 and CD11b, and negativity for Ly6G (CD45^+^Ly6G^−^F4/80^+^CD11b^+^) (gating strategy shown in supplementary materials Fig. S2). Of note, the term “BMMs” is referred to the whole population of M-CSF-stimulated BMCs/nBMCs and “BMMs” are considered as OCLs precursors.

Flow cytometry analysis of macrophages differentiated in the presence of M-CSF are shown in supplementary materials (Fig. S3).

### siRNA transfection

To examine the effect of HO-1 silencing in OCLs precursors, nBMCs-derived BMMs were replated in the presence of 30 ng/ml M-CSF and transfected with siRNA against HO-1 or scrambled control. One day after transfection fresh medium containing 50 ng/ml RANKL (to induce osteoclastogenesis) and 30 ng/ml M-CSF was added. After 3 days of incubation OCLs markers were analyzed by qPCR.

nBMCs-derived BMMs (or RAW264.7) pretreated with RANKL were used as early stage OCLs. To examine the effect of HO-1 silencing in early-stage OCLs, nBMCs-derived BMMs or RAW264.7 were replated with 50 ng/ml RANKL (and 30 ng/ml M-CSF in case of BMMs) and 24 h later (as early-stage OCLs) were transfected with siRNA against HO-1 or scrambled control. One day after transfection protein was collected for Western blot analysis or fresh RANKL-containing medium was added. After 3 days of incubation OCLs markers were analyzed by qPCR.

Cells cultured on 24-well plates at seeding density of 200 000 cells/400 μl α-MEM CM/well were transfected with Silencer Select siRNA against murine *Hmox1* or Silencer Select Negative Control (Thermo Fisher Scientific) using Lipofectamine RNAiMAX transfection reagent (Thermo Fisher Scientific) according to the manufacturer’s protocol. Briefly, 10 pmol of siRNA (0.5 μl) was diluted in OptiMEM to a final volume of 50 μl and then mixed with 50 μl of pre-diluted lipofectamine (3 μl of lipofectamine to 47 μl OptiMEM). The transfection mixture (100 μl) was incubated for 5 min at RT and then added dropwise to 400 μl of culture medium (α-MEM CM).

### Quantitative PCR

Total RNA isolation from cells cultured on 24-well plates at seeding density of 200 000 cells/well was performed by phenol-chloroform extraction. In brief, cells were washed with PBS, lysed with 400 μl of Fenozol (A&A Biotechnology), mixed with 100 μl of chloroform, vigorously mixed by vortexing for 60 sec and centrifuged (20 min, 10 000 × g, 4 °C). An upper aqueous phase was collected and subjected to isopropanol precipitation for at least 2 h at −20 °C. RNA pellets were resuspended in 12–25 μl of nuclease-free water.

Reverse transcription reaction was carried out on 0.5–1 μg of total RNA using oligo(dT) primers and RevertAid reverse transcriptase (Thermo Fisher Scientific), according to the vendor’s instructions in ProFlex PCR system thermocycler (Thermo Fisher Scientific) for 1 hour at 42 °C and subsequent 5 min at 95 °C. cDNA was stored at −20 °C. QPCR was performed in a mixture (15 μl) containing SYBR green Jumpstart Ready Mix (Sigma), 40 ng cDNA and specific primers as follows: *Hmox-1* (5′-CCTCACTGGCAGGAAATCATC -3′ and 5′-CCTCGTGGAGACGCTTTACATA-3′), *NFATC1* (5′-CTGCGGGAGCGGAGAAACTTTG-3′ and 5′-CTGGCAAGGCAGAGTGTGCTGT-3′), *CTSK* (5′-TGCAGCAGAACGGAGGCATTGA-3′ and 5′-GCCACTGCTCTCTTCAGGGCTT-3′), *EF2* (5′ GACATCACCAAGGGTGTGCAG-3′ and 5′-TCAGCACACTGGCATAGAGGC-3′) with the following cycling conditions: 10 min at 95 °C, 40 cycles: 30 sec at 95 °C, 1 min at 60 °C, and 45 sec at 72 °C, and final 10 min incubation at 72 °C. QPCR was performed in a StepOne-Plus real-time PCR system (Thermo Fisher Scientific). The EF2 housekeeping gene was used as a reference.

### Western blot

Cells were lysed in ice-cold RIPA buffer containing proteinase inhibitors, centrifuged (10 min, 8000 × g, 4 °C) and resuspended in RIPA buffer. Protein samples (50 µg each) and Page Ruler Prestained Protein Ladder (Thermo Fisher) were subjected to SDS-PAGE gel electrophoresis followed by a dry transfer of protein to a nitrocellulose membrane. Membranes were blocked in blocking buffer (TBS containing 0.1% Tween20 and 5% of fat-free milk) for 1 h at room temperature and then incubated overnight at 4 °C with antibodies against HO-1 (1:750, Enzo Life Sciences, ~32 kDa) and α-tubulin (1:1000, Calbiochem, ~55 kDa) as a loading control. After 30 min washing step in TBS containing 0.1% Tween20, HRP-conjugated secondary antibodies were used: anti-mouse IgG (1:5000, BD Biosciences) and anti-rabbit IgG (1:2000, Cell Signaling Technology). All antibodies were diluted in the blocking buffer. After the next washing step visualization was performed using SuperSignal West Pico chemiluminescence substrate (Pierce Biotechnology) according to the manufacturer’s instructions. Analysis was performed using ImageJ software.

### TRAP assessment

To recognize the active form of TRAP, TRAP 5b, TRAP staining (assessing intracellular enzyme activity) and TRAP ELISA (measuring the concentration of enzyme secreted by bone-resorbing OCLs) were done.

To assess the intracellular TRAP enzyme activity, BMMs were cultured on 96-well plates at seeding density of 100 000 cells/well. Specifically, BMCs-derived BMMs were stimulated with 100 ng/ml M-CSF and 100 ng/ml RANKL. Alternatively, BMCs-derived BMMs and nBMCs-derived BMMs were replated and cultured in the presence of 30 ng/ml M-CSF and 50 ng/ml RANKL. As a control, cells stimulated exclusively with M-CSF were used. After incubation cells were fixed and at the end of experiment TRAP (TRAcP 5b) was detected using Acid Phosphatase, Leukocyte (TRAP) Kit (Sigma-Aldrich) according to vendor’s protocol. TRAP-positive cells with 3 or more nuclei were counted using the Olympus IX81 microscope (Olympus).

The concentration of TRAP enzyme in the plasma of HO1^−/−^ and HO-1^+/+^ animals and media collected from the cells (cultured as described for TRAP staining) was measured using a MouseTRAP™ (TRAcP 5b) ELISA Immunoassay kit (Sigma-Aldrich) according to the manufacturer’s protocol. Briefly, 100 μl of anti-mouse TRAP antibody was added to the plate coated with anti-rabbit IgG antibodies and incubated for 1 h with shaking (850 rpm, RT). The plate was then rinsed 3 times with a washing buffer (250 μl) followed by the addition of plasma or culture media (25 μl), 0.9% NaCl (75 μl) and the release reagent (25 μl), and incubated for 1 h with shaking (850 rpm, TP). After this time the substrate solution (100 μl) was added for 2 h at 37 °C. After stopping the reaction by adding 25 μl of 1 M NaOH, the absorbance was measured at λ = 405 nm using the Infinite® 200 PRO reader (Tecan). TRAP concentration was assessed based on the standard curve.

#### CTX-1 ELISA

The concentration of C-telopeptide of type 1 collagen (CTX-1), collagen fragments released upon degradation of Type I collagen by osteoclasts, were assessed in the plasma of HO1^−/−^ and HO-1^+/+^ animals by RatLaps EIA according to manufacturer’s instructions. CTX-1 concentration was assessed based on the standard curve.

### Staining of actin structures

nBMCs-derived replated BMMs were cultured on 8-well chamber slides at seeding density of 100 000 cells/well. Subsequently cells were washed with PBS, fixed with 3.7% formaldehyde solution in PBS for 10 min, permeabilized with 0.5% Triton X-100 for 5 min and incubated with 100 nM Alexa Fluor 488-phalloidin (Cytoskeleton, Inc) for 30 min in the darkness. After washing with PBS, the cells were incubated with 100 nM DAPI in PBS for 30 sec and then covered with a fluorescent mounting medium (Dako) and cover slides. Pictures were taken with a Nikon Eclipse Ti microscope. Since it is difficult to distinguish small osteoclasts (<5 nuclei) with actin structures based on immunofluorescence staining, only multinucleate OCLs (>5 nuclei) with clusters of podosomes in the periphery of the cell were reliably distinguished and counted.

### Statistical analysis

All data are presented as mean of independent experiments ± standard error (SEM). Each experiment using RAW264.7 cells was done in duplicates. The mean of duplicate was considered as one independent experiment. In case of experiments using mice-derived primary cells or blood plasma each mouse was considered as one independent experiment. The number of independent experiments (n) is indicated in the appropriate figures legends. The highest n numbers are shown as dots on the appropriate graphs. Data were analyzed using unpaired Student’s t-test to compare two groups with normal distribution (checked with Shapiro-Wilk test when n > 6) or nonparametric Mann-Whitney test. Differences were accepted as statistically significant when p < 0.05.

## Results

### The effect of Hmox1 knockout in mice on osteoclasts precursors

**Figure 1:**
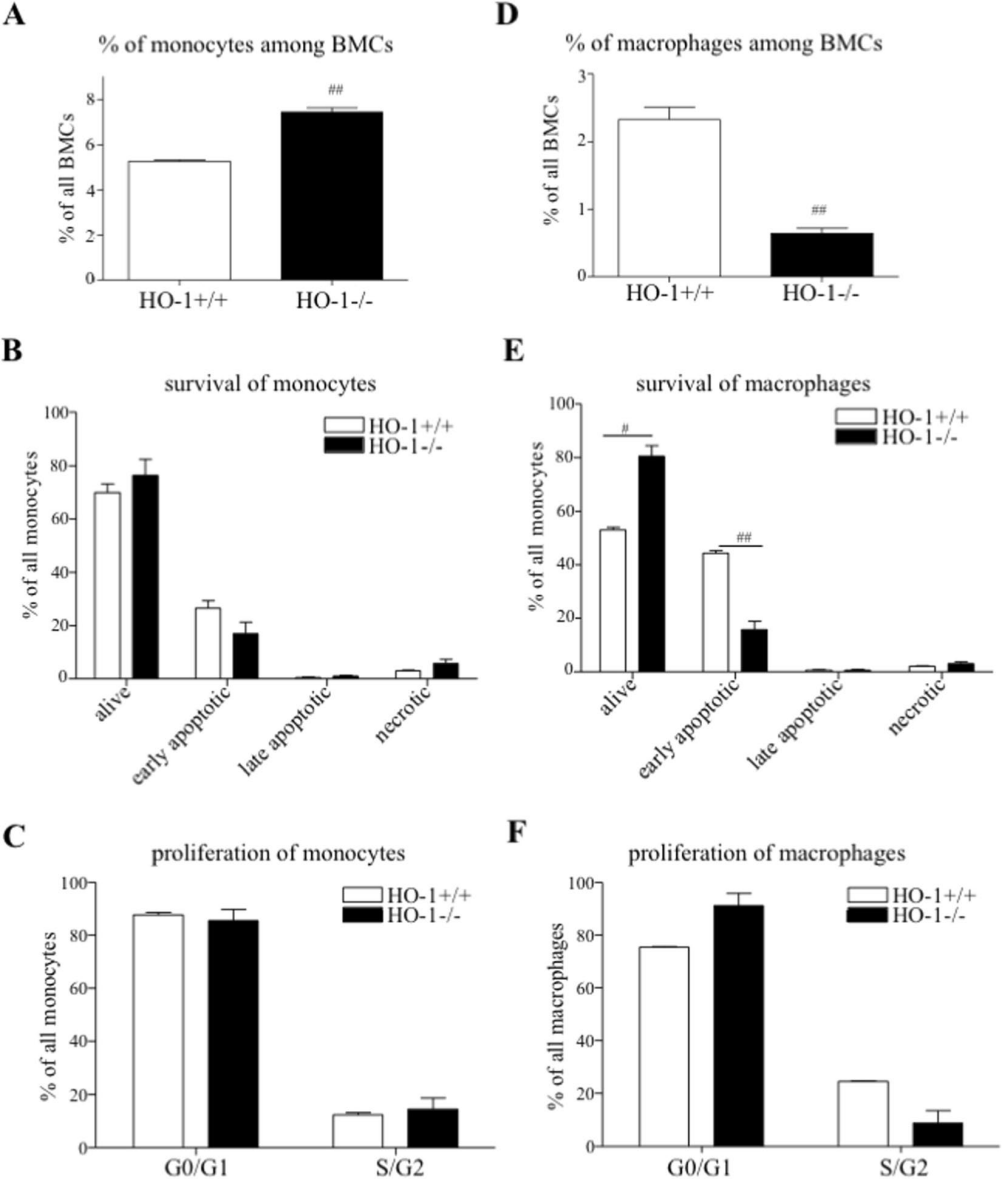
The effect of *Hmox1* knockout in mice on osteoclasts precursors in fresh bone marrow. Bone marrow was isolated from HO-1^−/−^ and HO-1^+/+^ mice. (**A**) The percentage of monocytes (CD45^+^Ly6G^−^Ly6C^+^CD11b^+^MHCII^low/−^F4/80^low/−^) among total BMCs, monocytes (**B**) survival and (**C**) proliferation (G0/G1 - non-proliferating cells, S/G2M - proliferating cells). (**D**) The percentage of macrophages (CD45^+^Ly6G^−^F4/80^+^CD11b^+^) among total BMCs, macrophages (**E**) survival and (**F**) proliferation (G0/G1 - non-proliferating cells, S/G2M - proliferating cells). Flow cytometry (n = 3). Each bar represents the mean ± SEM. ^#^p < 0.05, ^##^p < 0.01 vs. HO-1^+/+^.

Among the population of the freshly isolated BMCs and M-CSF-treated BMCs/nBMCs (BMMs), referred to as OCLs precursors, the percentage of strictly defined monocytes and macrophages was assessed. Among the whole population of BMCs in the fresh bone marrow of HO-1^−/−^ mice the percentage of monocytes defined as CD45^+^Ly6G^−^Ly6C^+^CD11b^+^MHCII^low/−^F4/80^low/−^ was higher in comparison to HO-1^+/+^ mice (7.43 ± 0.19 vs. 5.26 ± 0.06, respectively, Fig. 1A). However, HO-1^−/−^ and HO-1^+/+^ monocytes showed similar viability (Fig. 1B) and proliferation (Fig. 1C). A lower percentage of HO-1^−/−^ macrophages defined as CD45^+^Ly6G^−^F4/80^+^CD11b^+^ was detected in fresh bone marrow (0.64 ± 0.08 vs. 2.33 ± 0.18 of HO-1^+/+^, Fig. 1D). HO-1^−/−^ macrophages were more viable and contained a lower percentage of early apoptotic cells (Fig. 1E) (vs. HO-1^+/+^), while no significant influence of HO-1 deficiency on proliferation of macrophages was noticed (Fig. 1F). The gating strategy is shown in Supplementary Fig. S2.

**Figure 2:**
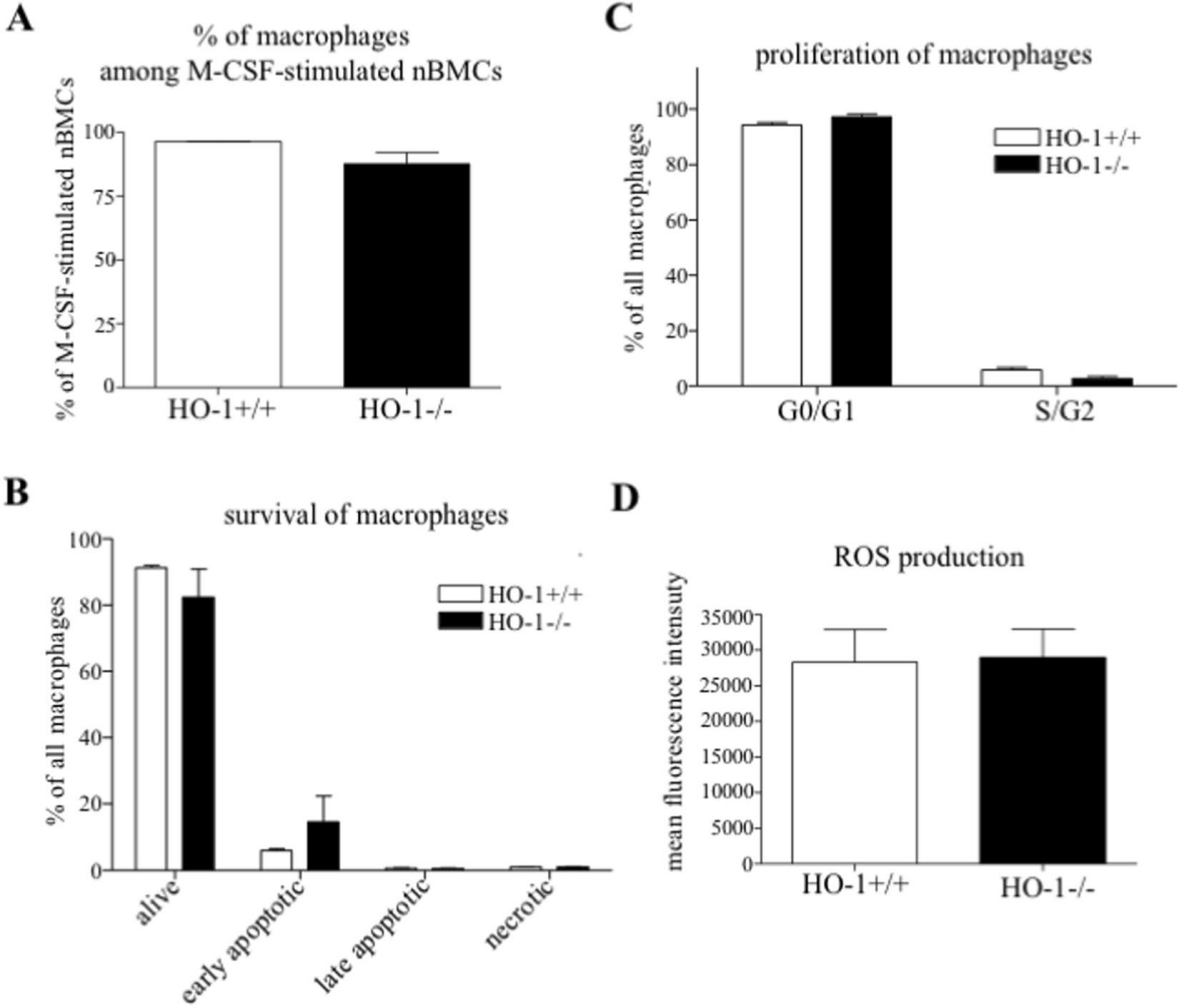
The effect of *Hmox1* knockout on the macrophages differentiation in the presence of M-CSF. Bone marrow was isolated from HO-1^−/−^ and HO-1^+/+^ mice. nBMCs were cultured with 50 ng/ml M-CSF for 3 days. (**A**) The percentage of macrophages (CD45^+^Ly6G^−^F4/80^+^CD11b^+^) among M-CSF-stimulated nBMCs, macrophages (**B**) survival, (**C**) proliferation (G0/G1 - non-proliferating cells, S/G2M- proliferating cells) and (**D**) ROS production. Flow cytometry (n = 3). Each bar represents the mean ± SEM.

After treatment with M-CSF towards macrophage overgrowth, no genotype-dependent difference was observed in the percentage of macrophage (Fig. 2A). Among macrophage population, the participation of alive, apoptotic cells (Fig. 2B) or proliferating cells (Fig. 2C), as well as the production of ROS (Fig. 2D), was comparable between the HO-1^−/−^ and HO-1^+/+^ groups (gating strategy shown in Supplementary Fig. S3). Thus, HO-1 deficiency seems not to affect macrophages differentiation in the presence of M-CSF.

### Both the lack of Hmox1 gene and HO-1 silencing in osteoclasts precursors decrease RANKL-induced expression of OCLs markers

**Figure 3:**
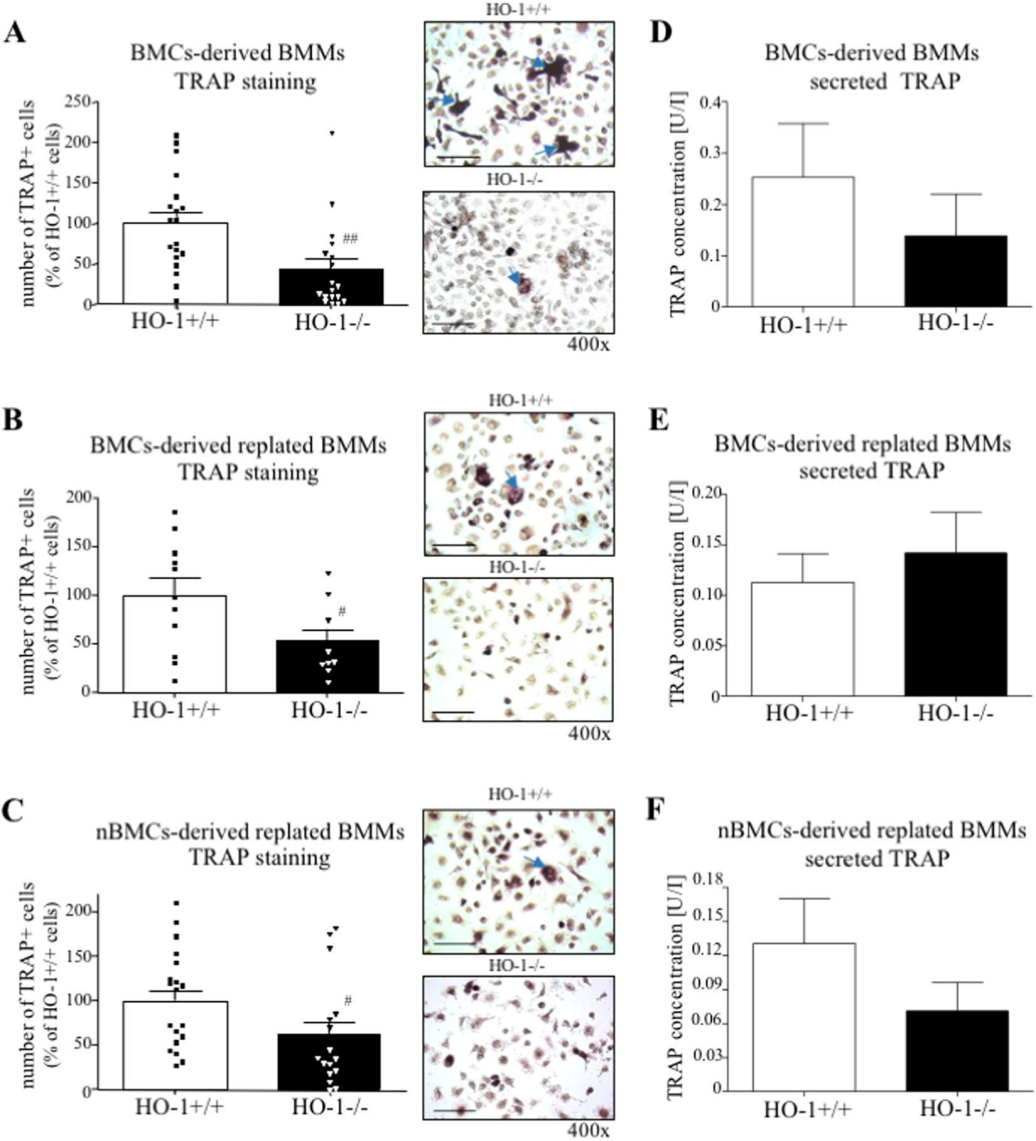
The effect of *Hmox1* knockout in osteoclasts precursors on TRAP+ cells formation. Bone marrow was isolated from HO-1^−/−^ and HO-1^+/+^ mice. (**A**,**D**) BMCs-derived BMMs were stimulated with 100 ng/ml RANKL in the presence of 100 ng/ml M-CSF for 3 days. Alternatively, (**B**,**E**) BMCs-derived BMMs and (**C**,**F**) nBMCs-derived BMMs were replated and cultured in the presence of 50 ng/ml RANKL and 30 ng/ml M-CSF for 3 days. (**A**,**B**,**C**) Quantitative analysis and representative pictures (magnification 400x, scale bar – 50 µm) of TRAP + cells (>2 nuclei). TRAP staining (n_A_ = 18–19, n_B_ = 9–11, n_C_ = 16–19). (**D**,**E**,**F**) TRAP concentration in the culture medium. TRAP ELISA (n = 5–6). Each bar represents the mean ± SEM. ^#^p < 0.05, ^##^p < 0.01 vs. HO-1^+/+^.

The hypothesis that HO-1 might be important for the RANKL-dependent induction of osteoclastogenesis in OCLs precursors was verified using three alternative experimental settings of the culture of primary cells. Overall, the lack of *Hmox1* gene attenuated differentiation of BMMs (BMCs-derived BMMs, BMCs-derived replated BMMs, and nBMCs-derived replated BMMs) towards OCLs (Fig. 3). Total BMCs HO-1^−/−^ cultured in the presence of M-CSF and directly stimulated with RANKL as BMCs-derived BMMs for 3 days showed lower number of TRAP + cells (2.31- fold decrease vs. HO-1^+/+^, Fig. 3A). Similar results were obtained when HO-1-deficient BMCs-derived BMMs (1.92- fold decrease vs. HO-1^+/+^, Fig. 3B) or nBMCs-derived BMMs (1.64- fold decrease vs. HO-1^+/+^, Fig. 3C), were replated prior to RANKL stimulation. Assessment of TRAP concentration in the culture medium reflected the number of TRAP + cells only to some extent (Fig. 3D,E,F, respectively).

**Figure 4:**
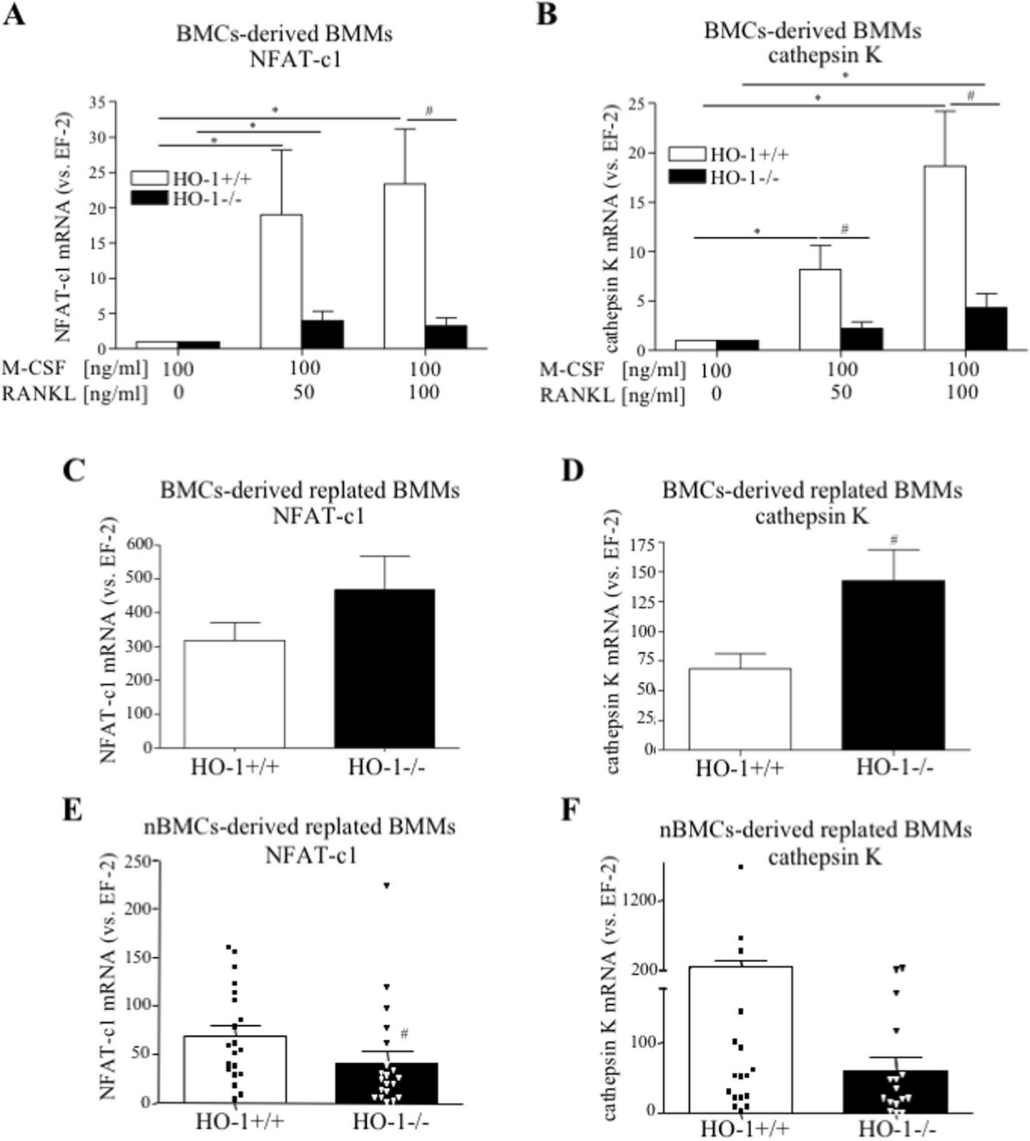
The effect of *Hmox1* knockout in osteoclasts precursors on OCLs-specific genes expression. Bone marrow was isolated from HO-1^−/−^ and HO-1^+/+^ mice. (**A**,**B**) BMCs-derived BMMs were stimulated with RANKL (50 ng/ml or 100 ng/ml) in the presence of 100 ng/ml M-CSF for 3 days. Alternatively, (**C**,**D**) BMCs-derived BMMs and (**E**,**F**) nBMCs-derived BMMs were replated and cultured in the presence of 50 ng/ml RANKL and 30 ng/ml M-CSF for 3 days. (**A**,**C**,**E**) NFAT-c1 and (**B**,**D**,**F**) cathepsin K relative expression (vs. EF-2). Quantitative PCR (n_A,B_ = 9–10, n_C,D_ = 7, n_E,F_ = 17–20). Each bar represents the mean ± SEM. *p < 0,05 vs. RANKL; ^#^p < 0.05 vs. HO-1^+/+^.

**Figure 5:**
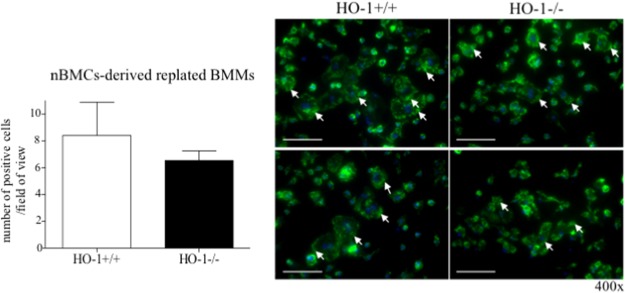
The effect of *Hmox1* knockout in osteoclasts precursors on the formation of actin structures. Bone marrow was isolated from HO-1^−/−^ and HO-1^+/+^ mice. nBMCs-derived BMMs were replated and cultured with 100 ng/ml RANKL for 5 days in the presence of 30 ng/ml M-CSF. Quantitative analysis (left) of multinucleated (>5 nuclei) cells with characterized actin structures and representative pictures (right, magnification 400x, scale bar –50 µm). Actin structures (green), nuclei (blue). White arrows indicate representative clusters of podosomes in the periphery of multinucleate cells. Immunofluorescence staining (n = 2–3, each from 12 fields of view). Each bar represents the mean ± SEM.

Moreover, the induction of OCLs markers, such as NFATc-1 and cathepsin K, in RANKL-stimulated BMCs-derived BMMs was diminished in the absence of HO-1 both at 50 ng/ml RANKL (4.74-fold for NFATc-1, 3.67-fold for cathepsin K vs. HO-1^+/+^) or 100 ng/ml RANKL (7.17-fold for NFATc-1, 4.32-fold for cathepsin K vs. HO-1^+/+^) (Fig. 4A,B). The lower concentration of RANKL was enough to induce the expression of OCLs markers independently of genotype (Fig. 4A,B). Interestingly, such changes in the level of NFAT-c1 and cathepsin K were not observed when BMCs-derived BMMs HO-1^−/−^ were replated and stimulated with RANKL (vs. HO-1^+/+^) (Fig. 4C,D). However, this effect of HO-1 deficiency was confirmed in RANKL-treated nBMCs-derived replated BMMs (1.65-fold decrease for NFATc-1 (p < 0.05), 3.43-fold decrease for cathepsin K vs. HO-1^+/+^, Fig. 4E,F). On the other hand, podosomes, peripheral actin-rich adhesive structures characteristic for OCLs, were formed in response to RANKL stimulation of BMMs independently of HO-1 presence (Fig. 5). No significant differences in the number of cells with detected actin structures were found between the HO-1^−/−^ and HO-1^+/+^ groups (Fig. 5), which suggests that HO-1 is dispensable for the formation of actin structures characteristic for OCLs.

**Figure 6:**
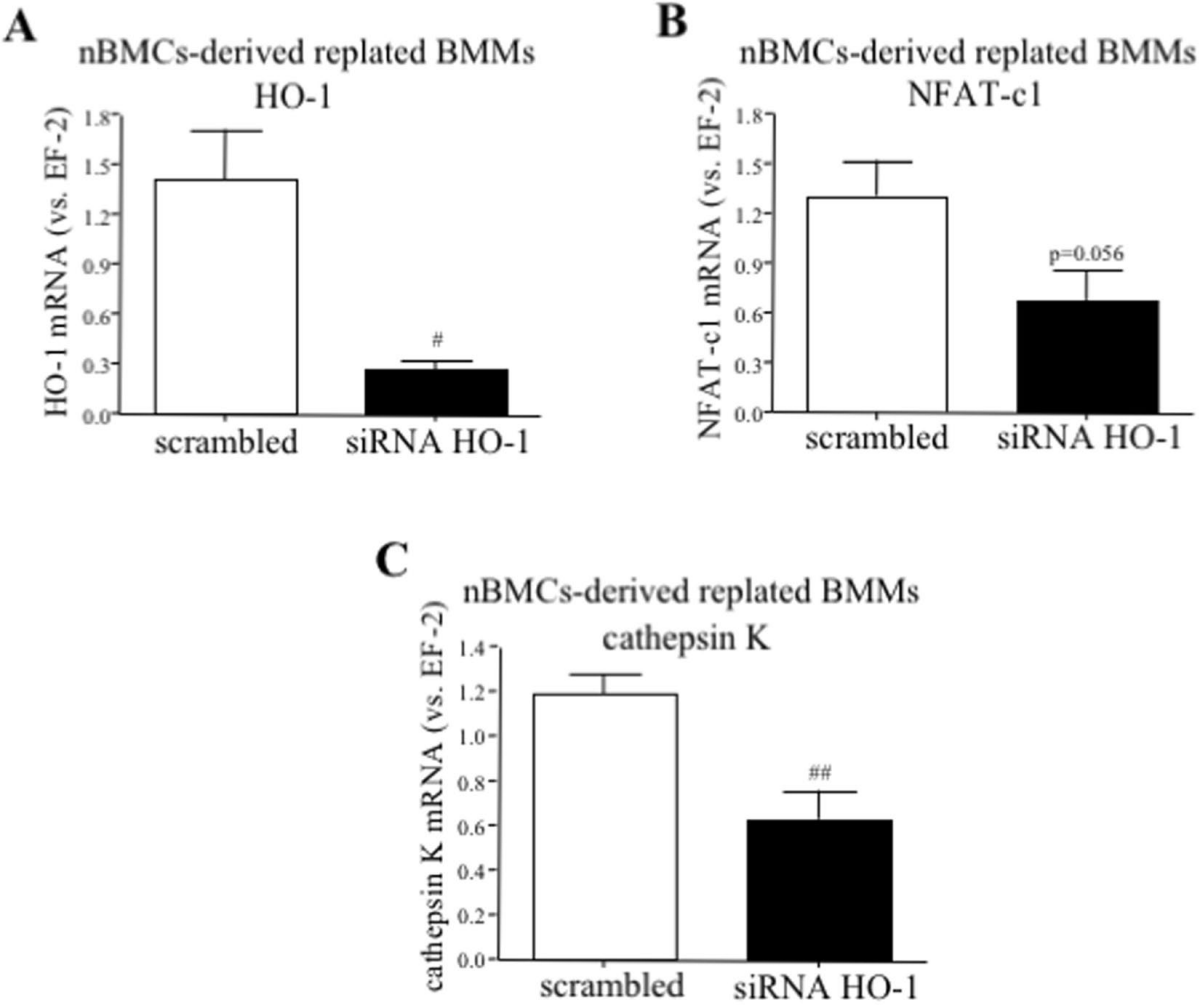
The effect of HO-1 silencing in osteoclasts precursors on the expression of OCLs markers. Bone marrow was isolated from HO-1^+/+^ mice. nBMCs-derived BMMs were replated and transfected with siRNA against HO-1 or scrambled control. One day after transfection fresh medium containing RANKL (50 ng/ml) and M-CSF (30 ng/ml) was added for the next 3 days. Quantitative PCR (n = 5). Each bar represents the mean ± SEM. ^#^p < 0.05, ^##^p < 0.01 vs. scrambled control.

siRNA transfection effectively reduced HO-1 mRNA expression in RANKL-stimulated nBMCs-derived BMMs (5.22-fold decrease vs. scrambled, Fig. 6A). Importantly, silencing of HO-1 lowered the level of both NFAT-c1 (1.96-fold decrease vs. scrambled, Fig. 6B) and cathepsin K (1.89-fold decrease vs. scrambled, Fig. 6C). In sum, HO-1 deficiency in OCLs precursors reduced RANKL-induced differentiation and the expression of OCLs markers.

### HO-1 is dispensable for early-stage osteoclasts

**Figure 7:**
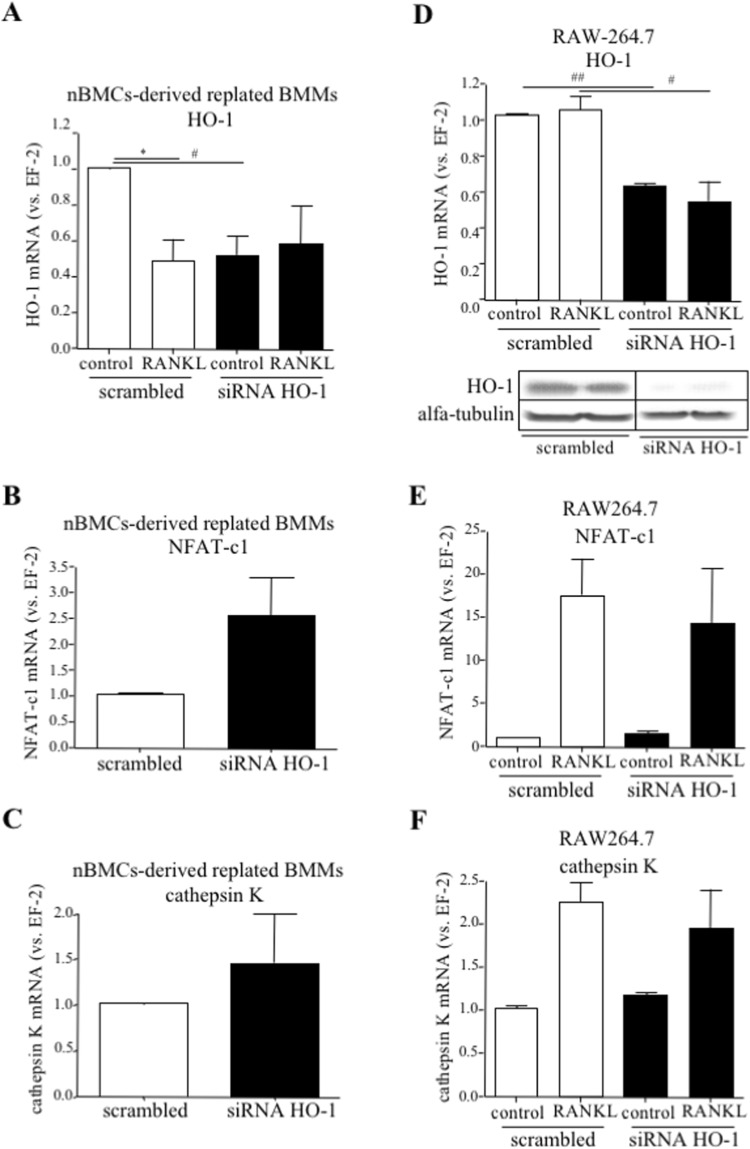
The effect of HO-1 silencing in early-stage OCLs on the expression of OCLs markers. (**A**–**C**) nBMCs HO-1^+/+^-derived BMMs and (**D**–**F**) RAW264.7 cells were replated with RANKL (50 ng/ml) and transfected with siRNA against HO-1 or scrambled control 24 h later (as early-stage OCLs). One day after transfection protein was collected or fresh RANKL-containing medium was added for the next 3 days. (**A**,**D**-upper panel) HO-1, (**B**,**E**) NFAT-c1 and (**C**,**F**) cathepsin K relative expression (vs. EF-2). Quantitative PCR (n = 3–4). (D-lower panel) HO-1 protein level. α-tubulin was used as a reference. Western blot (n = 2) – the grouping of gel/blot cropped from different parts of the same gel was performed (full-length blot presented in Supplementary Figure S4). Each bar represents the mean ± SEM. *p < 0,05 vs. RANKL; ^#^p < 0.05, ^##^p < 0.01 vs. scrambled control.

We confirmed the inhibitory effect of RANKL on HO-1 expression^29^ (2.06-fold decrease vs. control) as well as diminished mRNA level of HO-1 after siRNA transfection (1.94-fold decrease vs. scrambled) in early-stage OCLs (Fig. 7A). There was no further decrease of HO-1 mRNA upon combined siRNA and RANKL treatment (Fig. 7A). siRNA transfection did not also inhibit the expression of OCLs-specific genes such as NFAT-c1 or cathepsin K (Fig. 7B,C, respectively). Also in the RANKL-stimulated RAW264.7 cell line, silencing of HO-1 affected neither NFAT-c1 nor cathepsin K expression (Fig. 7D–F, Supplementary Fig. S4). Taking together, an inhibitory effect of HO-1 deficiency on the expression of OCLs markers was not reported when HO-1 expression was silenced in early stage-OCLs.

### The HO-1^−/−^ mice have higher level of TRAP in the plasma

**Figure 8:**
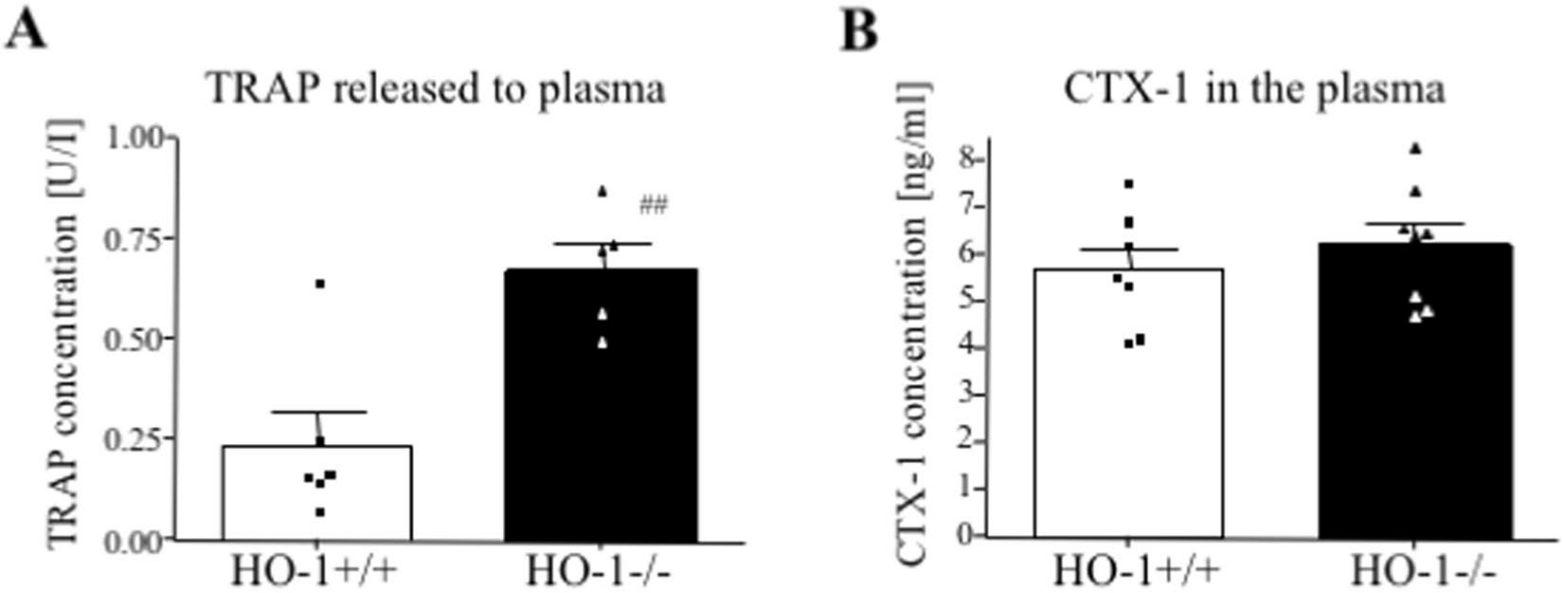
The effect of *Hmox1* knockout on osteoclasts number and activity *in vivo*. Plasma was isolated from HO-1^−/−^ and HO-1^+/+^ mice. (**A**) TRAP ELISA (n = 5–6). (**B**) CTX-1 ELISA (n = 7–8). Each bar represents the mean ± SEM. ^##^p < 0.01 vs. HO-1^+/+^.

In HO-1^−/−^ mice, a greater concentration of an active form of TRAP enzyme (isoform 5b) in the plasma was detected in comparison to HO-1^+/+^ mice (0.67 ± 0.06 vs. 0.24 ± 0.08, respectively, Fig. 8A). On the other hand, the level of CTX-1 was similar in the plasma of both genotypes (Fig. 8B).

### Hemin and CoPPIX inhibit OCLs-specific genes expression

**Figure 9:**
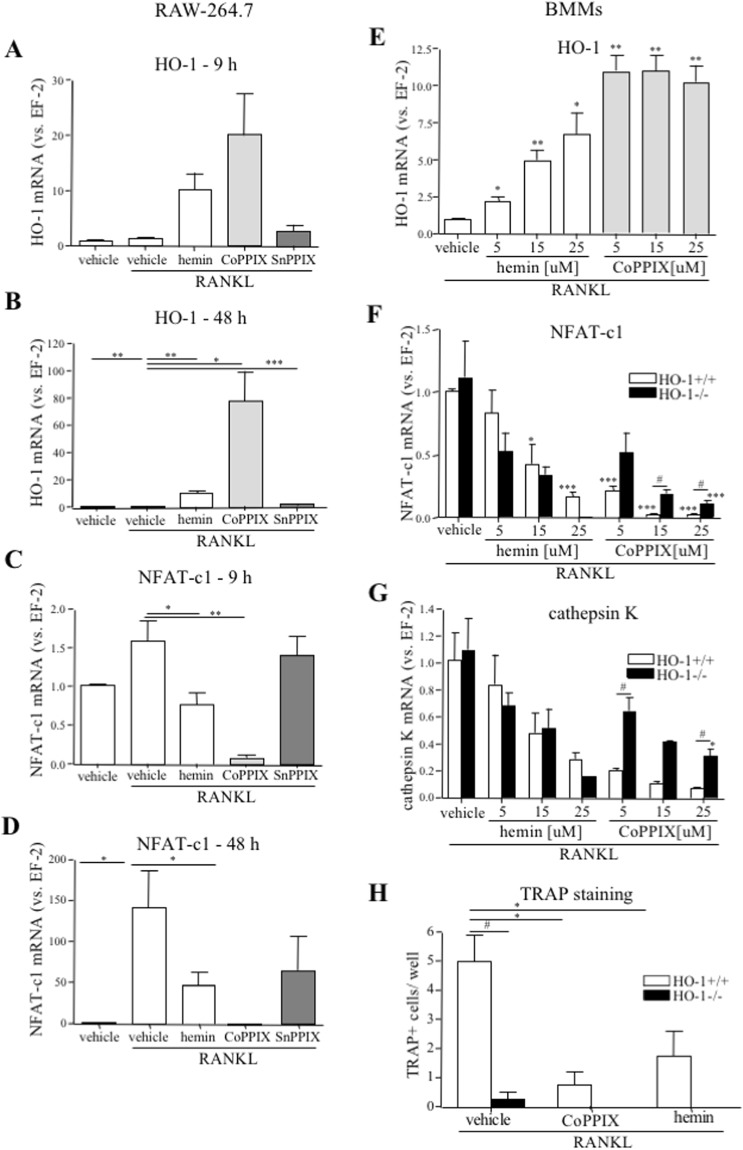
CoPPIX and hemin effect on osteoclasts-specific markers. (**A**–**D**) RAW264.7 were cultured with 50 ng/ml RANKL and 25 μM CoPPIX/hemin/SnPPIX or NaOH as a vehicle for (**A**,**C**) 9 h or (**B**,**D**) 48 h. (**A**,**B**) HO-1 and (**C**,**D**) NFAT-c1 relative expression (vs. EF-2). Quantitative PCR (n = 5–6). (**E**–**H**) Bone marrow was isolated from HO-1^−/−^ and HO-1^+/+^ mice. (**E**,**F**,**G**) nBMCs-derived BMMs were replated and cultured for 3 days with 50 ng/ml RANKL and 30 ng/ml M-CSF in the presence of 5, 15 and 25 μM CoPPIX/hemin or DMSO as a vehicle or (**H**) total BMCs-derived BMMs were stimulated with 100 ng/ml RANKL and 100 ng/ml M-CSF for 3 days in the presence of 25 μM CoPPIX/hemin or DMSO as a vehicle. (**E**) HO-1 (in HO-1^+/+^ cells), (**F)** NFAT-C1 and (**G**) cathepsin K relative expression (vs. EF-2). Quantitative PCR (n_E,F,G_ = 4). (**H**) Quantitative analysis of TRAP + cells (>2 nuclei). TRAP staining (n = 4). Each bar represents the mean ± SEM. *p < 0.05, **p < 0.05, ***p < 0.01 vs. vehicle; ^#^p < 0.05, ^###^p < 0.001 vs. HO-1^+/+^.

Hemin and CoPPIX treatment for either 9 h (p < 0.05) or 48 h increased the level of HO-1 in RANKL-stimulated RAW264.7 cells (Fig. 9A,B). Both pharmacological inducers of HO-1 decreased the level of NFAT-c1, enhanced by RANKL alone, with a stronger effect observed for CoPPIX (2.06- and 19.86-fold decrease after 9 h of hemin and CoPPIX treatment, respectively, Fig. 9C,D). Interestingly, no significant effect was detected in response to SnPPIX, an HO-1 inhibitor (Fig. 9C,D). CoPPIX and hemin stimulation of wild type RANKL-treated nBMCs-derived BMMs increased HO-1 expression already at a 5 μM concentration, with CoPPIX having a stronger effect than hemin (11.01 ± 1.05 vs. 2.25 ± 0.33, respectively, Fig. 9E). Both compounds concentration-dependently decreased the expression of NFAT-c1 (0.83 ± 0.18, 0.42 ± 0.16, 0.17 ± 0.03 at 5, 15 and 25 μM hemin, respectively; 0.21 ± 0.03, 0.03 ± 0.01, 0.02 ± 0.01 at 5, 15 and 25 μM CoPPIX, respectively, Fig. 9F) and cathepsin K (0.84 ± 0.22, 0.48 ± 0.15, 0.29 ± 0.05 at 5, 15 and 25 μM hemin, respectively; 0.21 ± 0.01, 0.11 ± 0.02, 0.07 ± 0.01 at 5, 15 and 25 μM CoPPIX, respectively, Fig. 9G). Moreover, the number of TRAP + cells was lower in response to hemin and CoPPIX (2.86- and 6.67-fold decrease, respectively, Fig. 9H). Importantly, the inhibitory effect of these agents towards NFAT-c1 or cathepsin K was also reported in HO-1^−/−^ cells (Fig. 9F,G). Strikingly, in response to hemin and CoPPIX no TRAP + cells were detected (Fig. 9H). In HO-1^−/−^ cells hemin treatment resulted in the lower level of OCLs markers than in the wild type counterparts (Fig. 9F,G,H). Of note, the stronger increase of NFATc-1 and cathepsin K in response to CoPPIX in HO-1^−/−^ cells (vs. HO-1^+/+^) was noticed but no such difference was detected in the case of TRAP (Fig. 9F,G,H).

### Nrf2 deficiency stimulates the expression of OCLs markers

Although the HO-1 effect in OCLs was shown to be more complex than previously suggested^28–30^, in accordance with previous results^33,34^, we confirmed the inhibitory effect of its upstream regulator, the Nrf2 transcription factor on OCLs markers expression.

**Figure 10:**
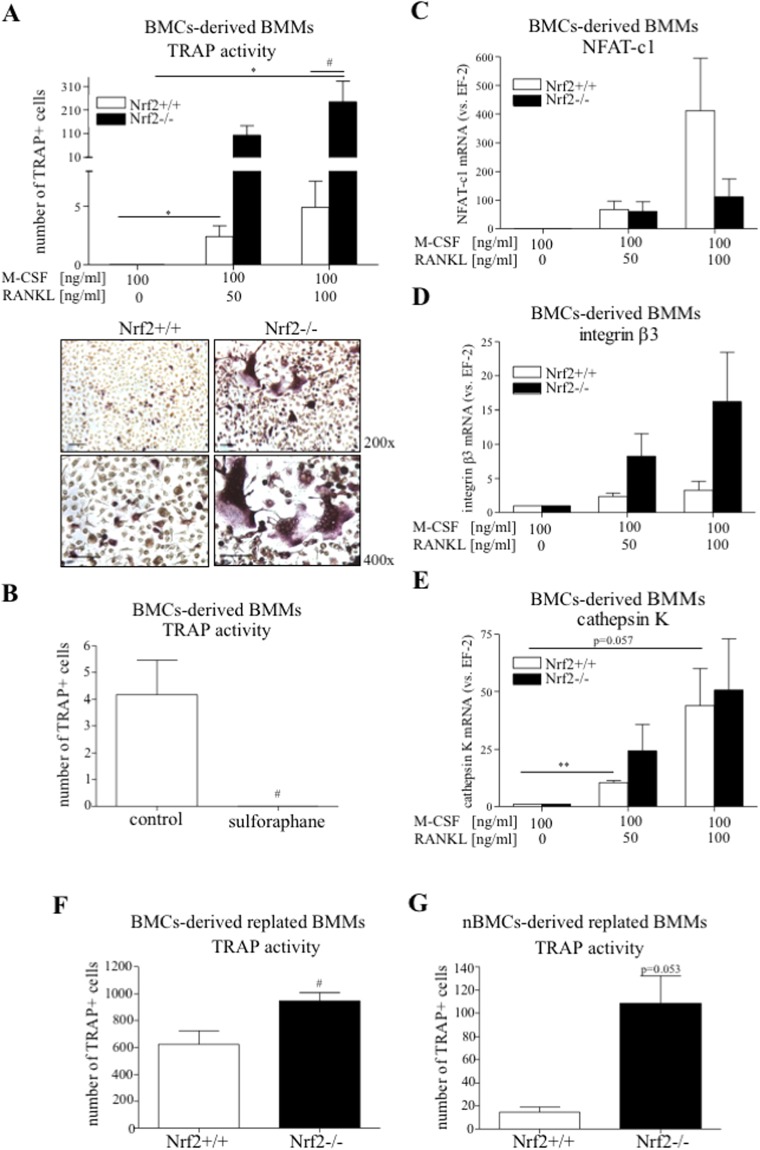
The effect of Nrf2 deficiency on OCLs markers expression. Bone marrow was isolated from Nrf2^−/−^ and Nrf2^+/+^ mice. (**A**–**E**) Total BMCs-derived BMMs were stimulated with RANKL (50 or 100 ng/ml where indicated), and 2.5 μM sulforaphane where indicated, for 3 days in the presence of 100 ng/ml M-CSF. Alternatively, (**F**) total BMCs-derived BMMs or (**G**) nBMCs-derived BMMs were replated and treated with RANKL (50 ng/ml) in the presence of M-CSF (30 ng/ml). (**A**,**B**) Quantitative analysis and representative pictures (magnification 200x -upper panel and 400x -lower panel, scale bar – 50 µm) of TRAP + cells. TRAP staining (n = 6). (**C**) NFAT-c1, (**D**) integrin β3 and (**E**) cathepsin K relative expression (vs. EF-2). Quantitative PCR (n = 4–6). (**F**,**G**) Quantitative analysis of TRAP + cells. TRAP staining (n = 3–4). Each bar represents the mean ± SEM. *p < 0.05 vs. RANKL; ^#^p < 0.05, ^##^p < 0.01 vs. control/Nrf2^+/+^.

Among BMCs-derived BMMs obtained from Nrf2^−/−^ mice, a higher number of TRAP+ cells in response to RANKL (49.73-fold increase vs. Nrf2^+/+^ at 100 ng/ml RANKL) (Fig. 10A) was detected. Additional treatment of wild type BMCs-derived BMMs with sulphoraphane, a known Nrf2 activator, gave no TRAP-positive signal (Fig. 10B). Although the level of NFAT-c1 was lower in RANKL-treated BMCs-derived BMMs Nrf2^−/−^ (vs. Nrf2^+/+^) (Fig. 10C), enhanced expression of integrin β3 was reported (Fig. 10D). No significant effect of Nrf2 deficiency was observed for cathepsin K (Fig. 10E). Importantly, when Nrf2-deficient BMCs-derived BMMs were replated and stimulated with RANKL, more TRAP + cells were detected in comparison to the wild type counterparts (1.52-fold increase vs. Nrf2^+/+^, Fig. 10F) as it was for nBMCs-derived BMMs Nrf2^−/−^ (7.41-fold increase vs. Nrf2^+/+^, Fig. 10G).

## Discussion

The importance of antioxidants as well as their upstream regulators in osteoclastogenesis has been reported and considered as a therapeutic approach towards destructive bone diseases. Among three fundamental mechanisms regulating the expression of cytoprotective enzymes including Nrf2-, FOXO-, and sirtuin-dependent pathways, each was reported to inhibit OCLs differentiation and bone destruction via attenuation of intracellular ROS signalling^17^. Recent studies have also suggested an inhibitory effect on osteoclastogenesis of HO-1, the Nrf2 downstream target^28–30^. However, none of those, aimed at a comparison of the effect of HO-1 deficiency in OCLs precursors, i.e., before stimulation by RANKL, versus early-stage OCLs, i.e., after stimulation by RANKL. We hypothesized that HO-1 involvement at different stages of the osteoclastogenesis might vary. Since HO-1 seems to be critical for the differentiation of OCLs progenitors, stimulating myeloid lineage differentiation and controlling macrophage condition^35^, it might be important not only as a ROS scavenger attenuating OCLs differentiation but for the induction of osteoclastogenesis as well. Here, we confirmed the inhibition of the expression of OCLs markers by Nrf2. We showed that HO-1 is crucial for the response of OCLs precursors to RANKL but it is dispensable in early-stage OCLs.

The myeloid-monocytic lineage of the bone marrow is recognized as the origin of macrophages and, at a later stage, OCLs. We showed that HO-1 knockout results in the lower number of macrophages in murine bone marrow. This agrees with the previous findings by Wegiel *et al*. pointing at the importance of HO-1/CO in the ability of myeloid progenitors to differentiate toward macrophages^35^. On the other hand, in our experimental setting, HO-1 deficiency seems not to affect macrophages differentiation in the presence of M-CSF. Nonetheless, to check if there is a role of HO-1 in the induction of osteoclastogenesis, the effect of HO-1 deficiency in OCLs precursors towards differentiation to OCLs was examined.

We showed that both, the lack of *Hmox1* gene and HO-1 silencing in primary macrophages decreased RANKL-induced differentiation towards OCLs. Because the preliminary results with the BMCs-derived BMMs were unexpected in light what was known before on the effect of HO-1 in osteoclastogenesis, we decided to check different populations of BMCs-derived cells and strategies for differentiation towards OCLs. Neither when using BMCs-derived BMMs nor BMCs-derived replated BMMs or nBMCs-derived replated BMMs did we confirm the inhibitory effect of HO-1 on the RANKL-induced expression of OCLs markers, which were observed by other groups^29,30^. Instead, all three cell types gave similar results of TRAP staining (in response to RANKL) which is considered as an important cytochemical marker of OCLs. This proves that the effect of HO-1 deficiency observed by our group is not specific to one type of OCLs precursors/experimental setting. In addition, based on the obtained results, where no effect of HO-1 was detected on the number and viability of BMCs-derived macrophages, the inhibitory influence of HO-1 deficiency on the RANKL-induced expression of OCLs markers might not be related to the condition of precursors. Further investigations are necessary in order to find the underlying mechanism.

In the studies of other groups, a population of nBMCs was usually used to obtain BMMs. Data published so far pointed at the inhibitory effect of HO-1 in OCLs precursors on their differentiation to OCLs^29,30^. Namely, the induction of HO-1 with hemin or curcumin in BMMs or RAW-D macrophages inhibited osteoclastogenesis and suppressed the release of OCLs cytokine, high mobility group box 1 (HMGB1)^29^. Moreover, suppression of HO-1 by siRNA in RAW-D cells promoted the activation of HMGB1^29^. Accordingly, more TRAP+ multinucleated cells were detected in RANKL-stimulated BMMs derived from HO-1^−/−^ mice (vs. HO-1^+/+^)^30^. The effect of HO-1 was correlated with heme metabolites^29,31^. Both bilirubin and CORM2 (a CO donor), but not iron, inhibited RANKL-mediated HO-1 suppression and consequently blocked osteoclastogenesis^29^. Specifically, HO-1/CO axis suppressed RANKL-induced osteoclastic differentiation by inhibiting redox-sensitive NF-κB activation^31^. In our hands, the data based on nBMCs-derived BMMs was not convincing, so the experiment was repeated several times (high “n” number) to verify the final effect of HO-1 deficiency in those cells.

The different strain of the mice used for isolation or details in experimental protocol might lay at the root of observed differences between ours and other groups. One needs to also remember that some stromal contamination of BMCs may affect the results and the used populations of so called BMMs should not be considered as a homogenous culture of macrophages.

In addition, we demonstrated that commonly used pharmacological inducers of HO-1, CoPPIX, and hemin, despite the effective increase of HO-1 level, may evoke HO-1-independent effects on OCLs markers. This phenomenon was not shown in previous studies. For example, Yashima *et al*. reported that CoPPIX inhibits RANKL-induced OCLs formation in a dose-dependent manner through blocking multiple signalling pathways such as Akt, ERK, p38 MAPK, JNK, and IκBα^38^. However, no direct evidence was demonstrated in that study [20] or in the other reports regarding an involvement of HO-1 in CoPPIX- or hemin-dependent repression of osteoclastogenesis^28,29,38^. In our model, the inhibitory effect of CoPPIX (5–25 μM) and hemin (5–25 μM) towards OCLs markers were also reported in HO-1^−/−^ cells. Moreover, in HO-1^−/−^ cells hemin treatment (25 μM) resulted in the lower level of OCLs markers. This may be related to sensitivity of HO-1-deficient cells to cytotoxicity caused by hemin^39^. The effect of CoPPIX seems to be more complicated and needs further investigation. Thus, previous conclusions on the inhibitory effect of HO-1 based only on the use of these pharmacological agents should be reconsidered.

Interestingly, we showed that HO-1 is dispensable when osteoclastogenesis is already induced (in early-stage OCLs), which has not been explored so far. In addition, an increased plasma level of TRAP (TRAcP 5b) was detected in HO-1^−/−^ mice. This may reflect the increased number of active bone-resorbing OCLs in such animals. However, the plasma level of CTX-1, a marker of bone resorption, was similar in both genotypes. Thus, the inhibition of the activity of mature OCLs *in vivo* by HO-1 need to be further verified. Importantly, Ke *et al*. reported a decrease in the bone mass and elevated serum CTX-1 and TRACP5b levels in HO-1^−/−^ mice under physiological conditions^30^. At the same time, there appeared to be no significant difference in bone formation as indicated by serum PINP and osteocalcin levels^30^.

The explanation of observed differences of HO-1 in OCLs precursors versus early-stage OCLs, could be related to the self-amplification of the NFAT-c1 transcription factor^11^. It could be based on the observation that HO-1 deficiency in OCLs precursors inhibited the expression of NFAT-c1 and ultimately reduced the abundance of differentiated cells. On the other hand, when HO-1 deficiency occurred after RANKL-induced differentiation, NFAT-c1 signalling was already induced and probably sustained by NFAT-c1 auto-amplification^11^. The effect of HO-1 deficiency *in vivo* on the number of mature OCLs is in agreement with previous studies^30^ and its antioxidant actions.

In conclusion, the HO-1 is crucial for the response of OCLs precursors to RANKL and the induction of OCLs markers, but it seems to be dispensable in RANKL-pre-stimulated cells considered as early-stage OCLs. However, *in vivo* HO-1 appears to inhibit osteoclastogenesis. This shows that caution should be given to suggestions on the use of HO-1 as a target for the treatment of skeletal diseases. The effects of HO-1 in bone remodelling requires further analysis and its multi-faced action in those processes should be considered.

## Electronic supplementary material

Supplementary figures and legends
