# Supplementary material for: Various roles of heme oxygenase-1 in response of bone marrow macrophages to RANKL and in the early stage of osteoclastogenesis
Source: Sci Rep. 2018 Jul 17;8:10797. doi: 10.1038/s41598-018-29122-1 (PMC6050304; doi:10.1038/s41598-018-29122-1)
Supplement: Supplementary file 1 — Supplementary figures and legends [file 41598_2018_29122_MOESM1_ESM.doc]

SUPPLEMENTARY INFORMATION

Various roles of heme oxygenase-1 in response of bone marrow macrophages to RANKL and in the early stage of osteoclastogenesis

Urszula Florczyk-Soluch^1*^, Ewelina Józefczuk^1^, Jacek Stępniewski^1^, Karolina Bukowska-Strakova^3^, Mateusz Mendel^1^, Monika Viscardi^1^, Witold Norbert Nowak^1^, Alicja Józkowicz^1^, Józef Dulak^1,2*^

^1^Department of Medical Biotechnology, Faculty of Biochemistry, Biophysics and Biotechnology, Jagiellonian University, Krakow, Poland

^2^ Kardio-Med Silesia, Zabrze, Poland

^3^ Department of Clinical Immunology, Institute of Pediatrics, Jagiellonian University Medical College, Krakow, Poland

* Corresponding authors:

Prof. Jozef Dulak, PhD, DSc, Department of Medical Biotechnology, Faculty of Biochemistry, Biophysics and Biotechnology, Jagiellonian University, Gronostajowa 7, 30-387 Krakow, Poland; phone: +48-12-664-63-75; fax: +48-12-664-69-18; email: jozef.dulak@uj.edu.pl

Urszula Florczyk-Soluch, PhD, Department of Medical Biotechnology, Faculty of Biochemistry, Biophysics and Biotechnology, Jagiellonian University, Gronostajowa 7, 30-387 Krakow, Poland; phone: +48-12-664-63-98; fax: +48-12-664-69-18; email: urszula.florczyk@uj.edu.pl

**
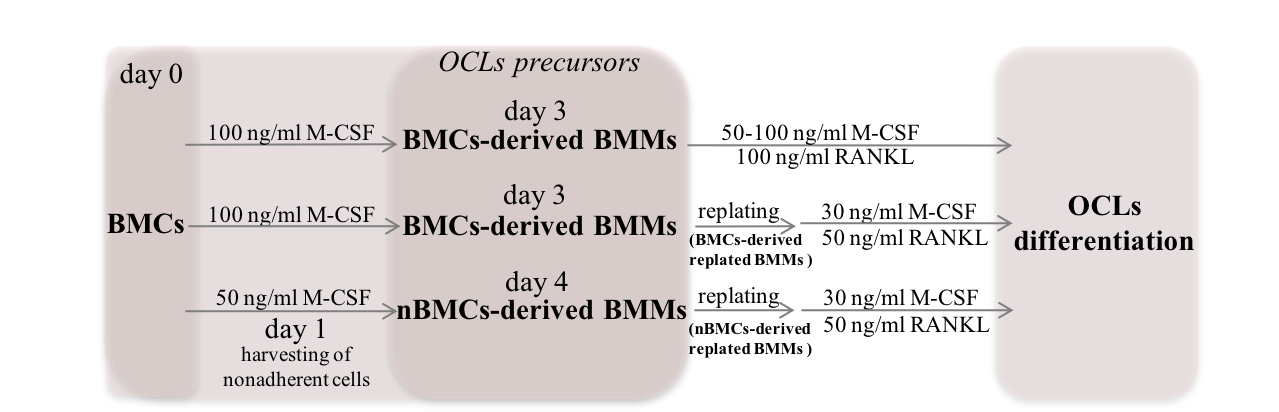
**

**Figure S1. Simplified scheme of cell culture experimental settings**

**
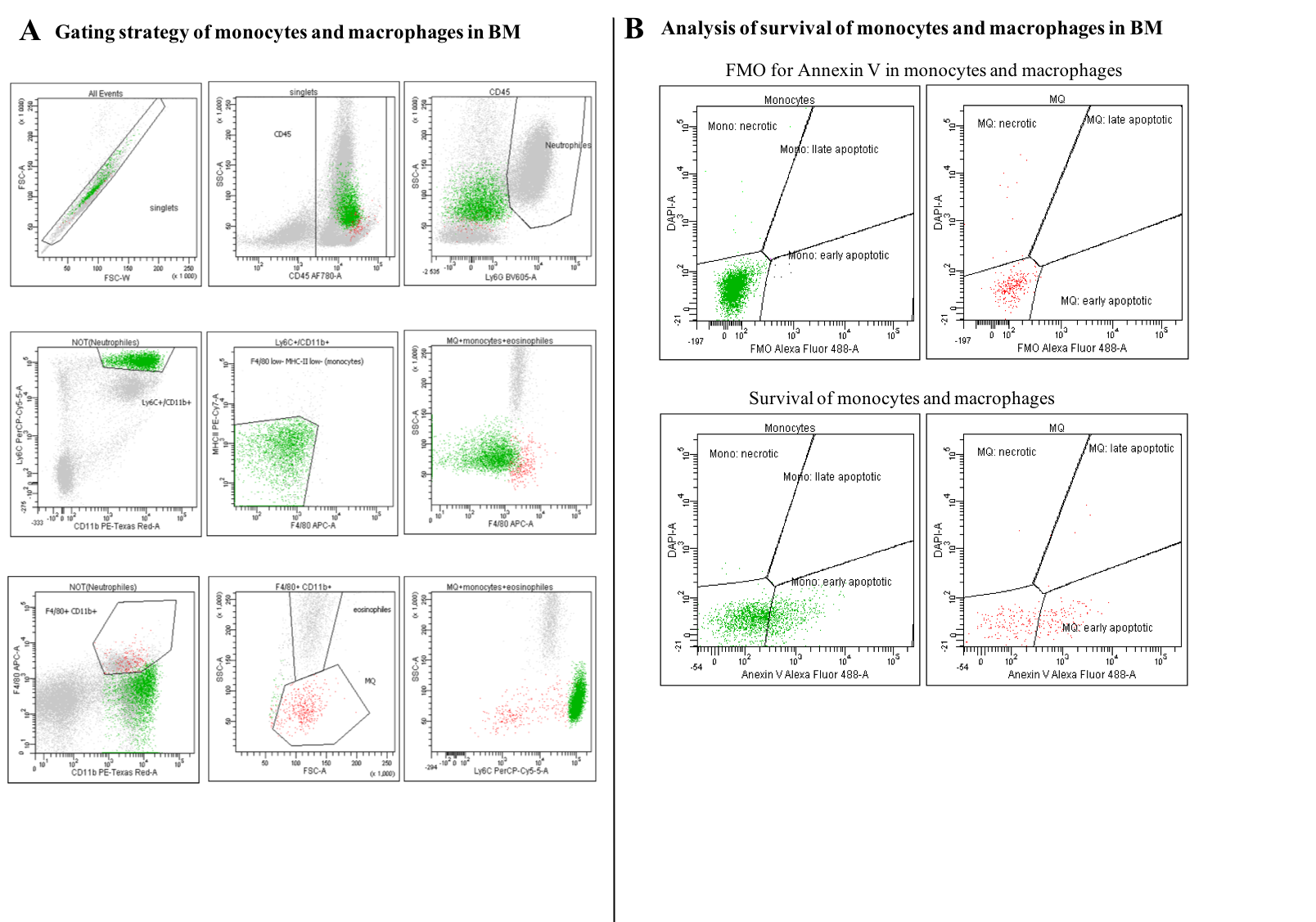
**

**Figure S2**. **Gating strategy of monocytes and macrophages in BM. (A)** Global gating strategy to distinguish monocytes and macrophages (MQ) in bone marrow (BM). In the first step, doublets were excluded, then CD45 positive cells were taken into further analysis. Next, based on Ly6G expression and SSC values, neutrophiles were excluded. Both monocytes and macrophages were initially identified as CD45 positive and Ly6G negative. Then, in one approach, monocytes were identified based on high Ly6C and CD11b expression and low to negative MHCII and F4/80 expression (CD45^+^Ly6G^-^Ly6C^+^CD11b^+^MHCII^low/-^F4/80^low/-^). In the second approach macrophages were identified based on positivity for F4/80 and CD11b (CD45^+^Ly6G^-^F4/80^+^CD11b^+^). Finally, macrophages were back gated based on SSC and FSC values to exclude eosinophils. (B) Analysis of survival of monocytes and macrophages in BM. For proper Annexin V gating, FMO controls were applied.

**
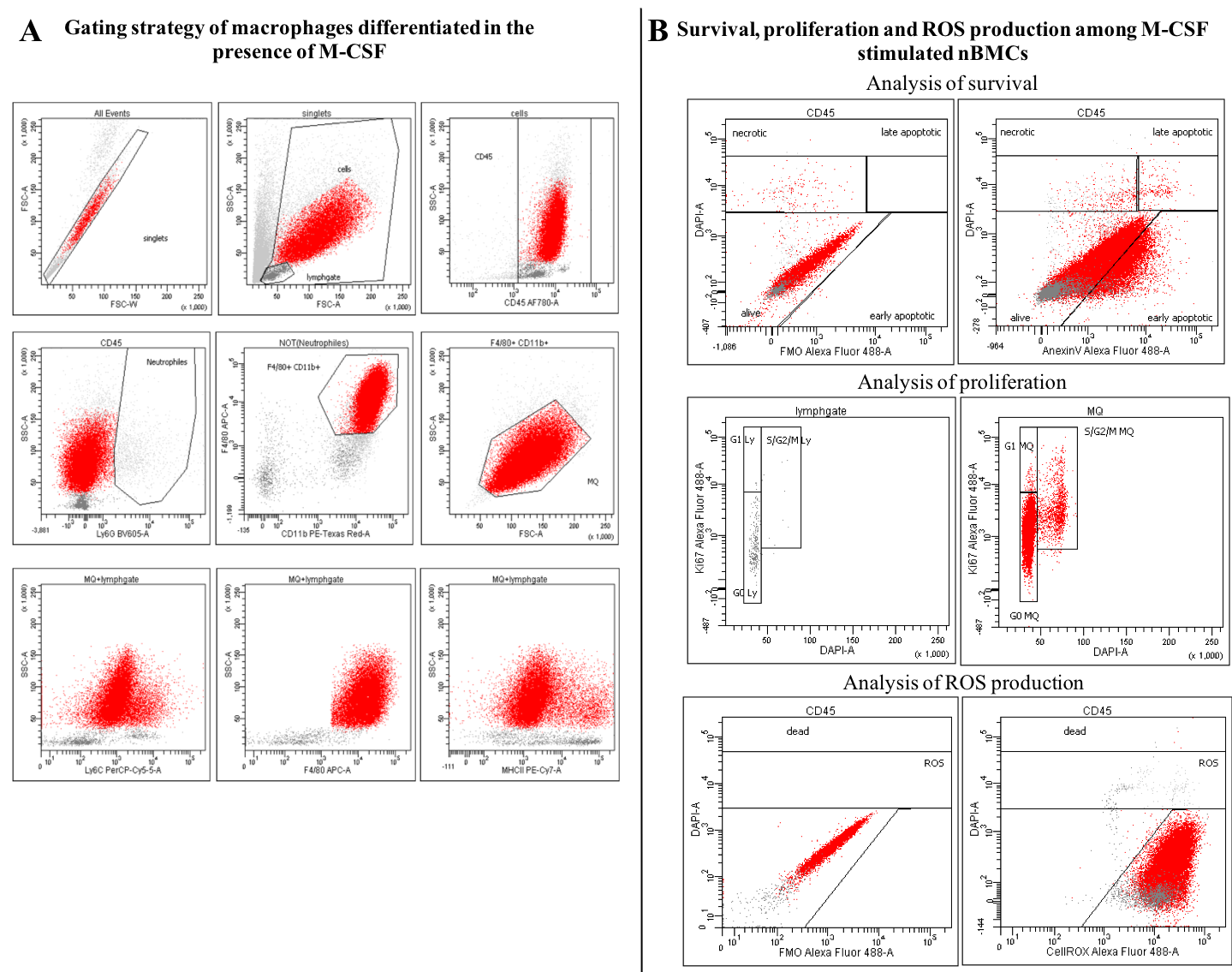
**

**Figure S3**. **Gating strategy of macrophages differentiated in the presence of M-CSF.** (A) Gating strategy of macrophages differentiated in the presence of M-CSF was analogous as in case of bone marrow (CD45^+^Ly6G^-^F4/80^+^CD11b^+^). The relatively small, non-granular cells, represented residual lymphocyte subpopulations, which served as internal staining control. (B) Analysis of survival, proliferation and ROS production, with appropriate controls (FMO for Annexin V and ROS production and in-sample control of residual lymphocytes in case of Ki67 staining).

**
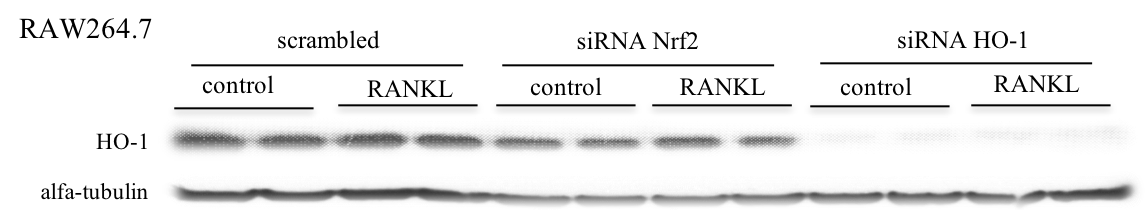
**

**Figure S4. HO-1 silencing in RAW264.7.** RAW264.7 cells were seeded with RANKL (50 ng/ml) and transfected with siRNA against HO-1 or scrambled control 24 h later (as early-stage OCLs). One day after transfection protein was collected. HO-1 protein level. α-tubulin was used as a reference. Western blot (n=2)
